# Supplementary material for: Lucanthone, Autophagy Inhibitor, Enhances the Apoptotic Effects of TRAIL through miR-216a-5p-Mediated DR5 Upregulation and DUB3-Mediated Mcl-1 Downregulation
Source: Int J Mol Sci. 2021 Dec 21;23(1):17. doi: 10.3390/ijms23010017 (PMC8744864; doi:10.3390/ijms23010017)
Supplement: Supplementary file 1 [file ijms-23-00017-s001.zip › f-original data.pdf]

Figure 1A

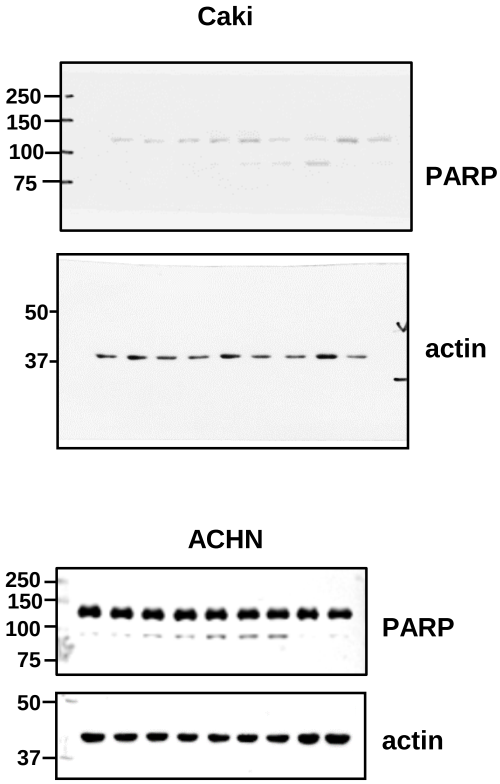

Figure 1E

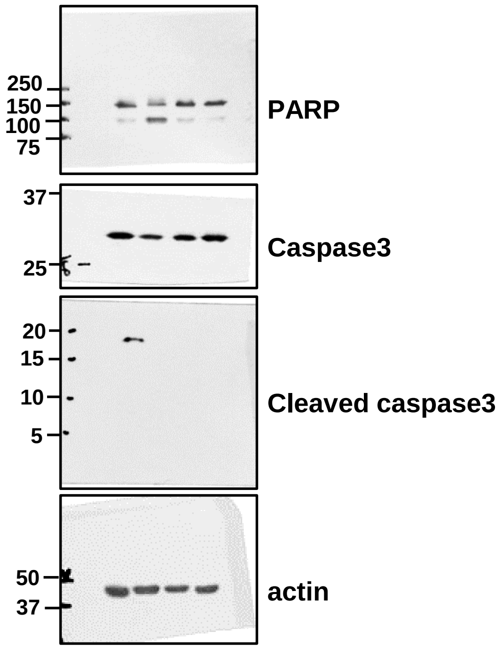

Figure 2A

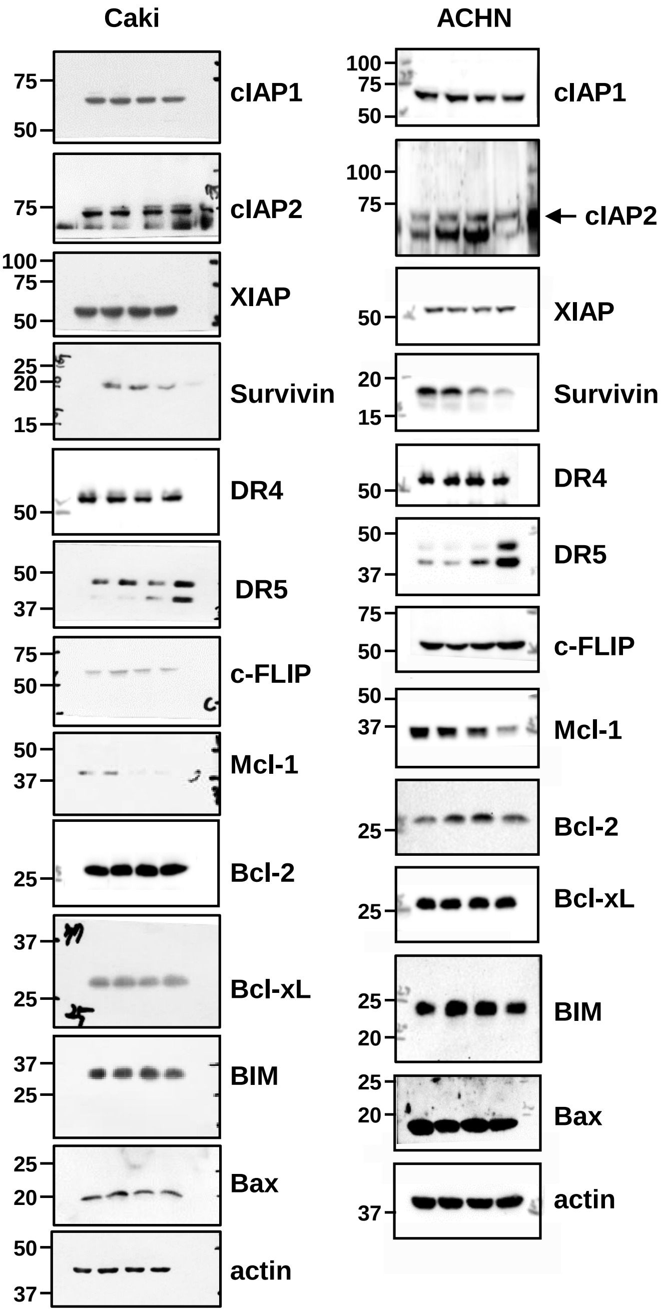

Figure 2B

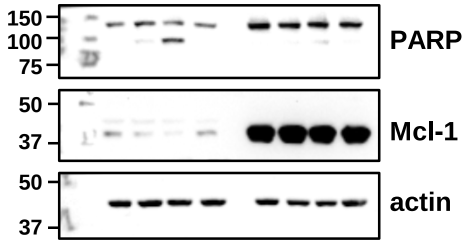

Figure 2C

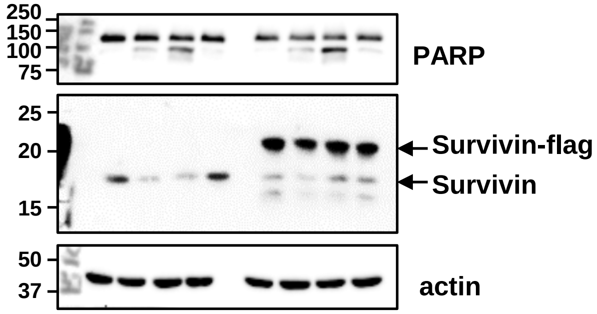

Figure 3A

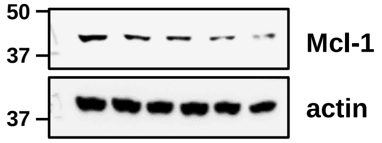

Figure 3C

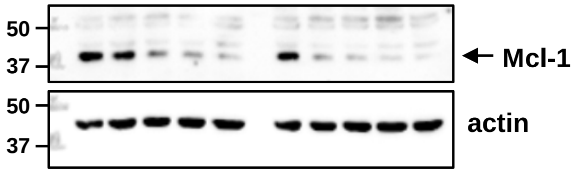

Figure 3D

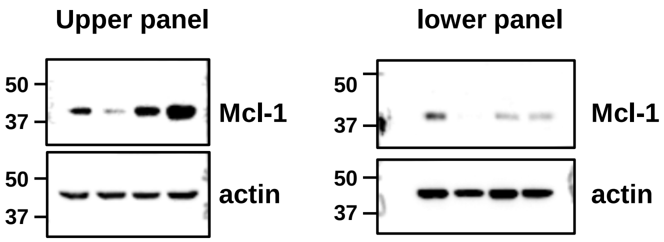

Figure 4A

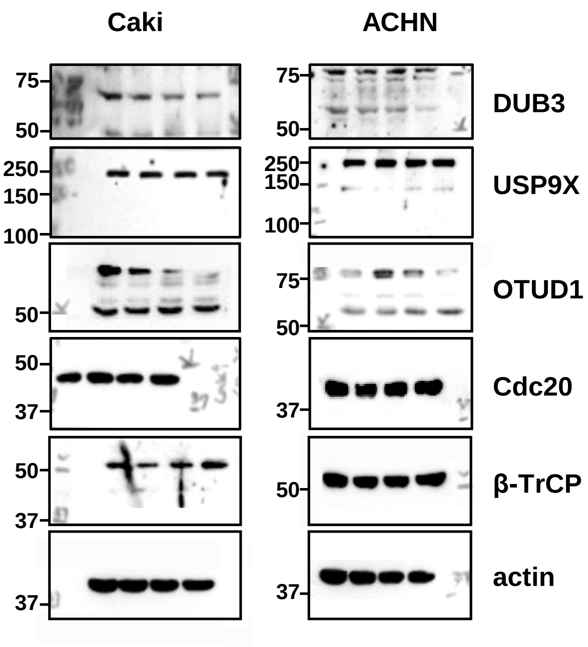

Figure 4B

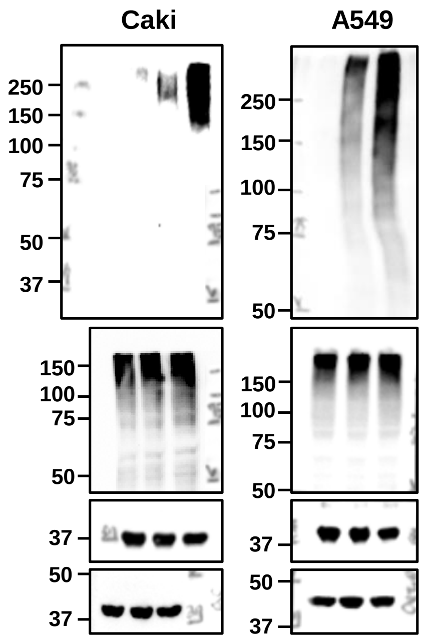

Figure 4C

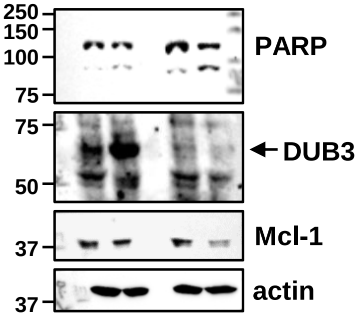

Figure 4D

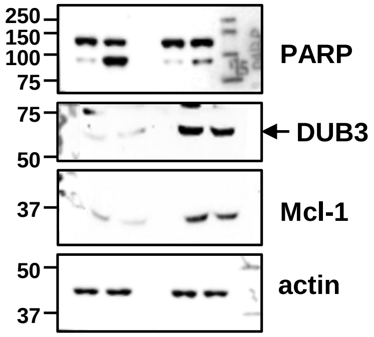

Figure 5E

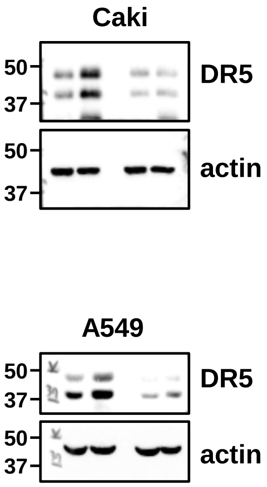

Figure 5F

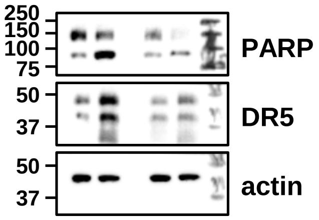

Figure 6A

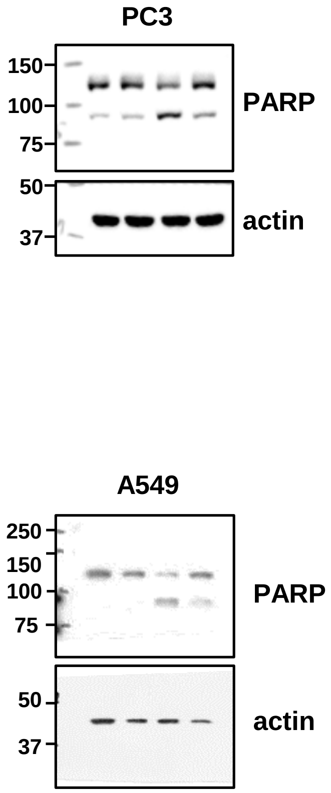

Figure 6B

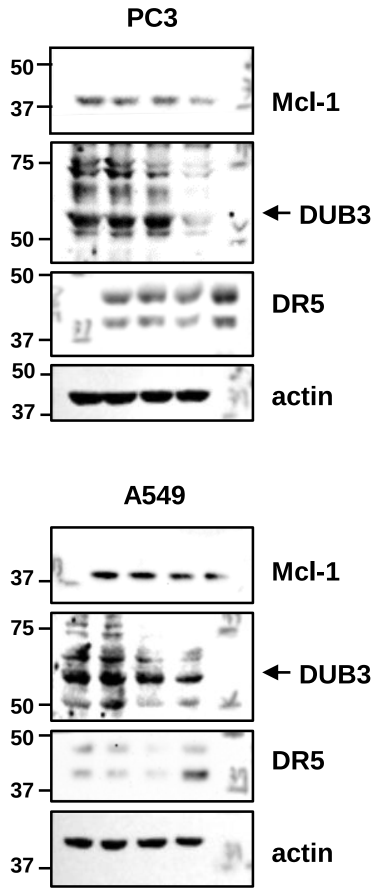

Figure S2B

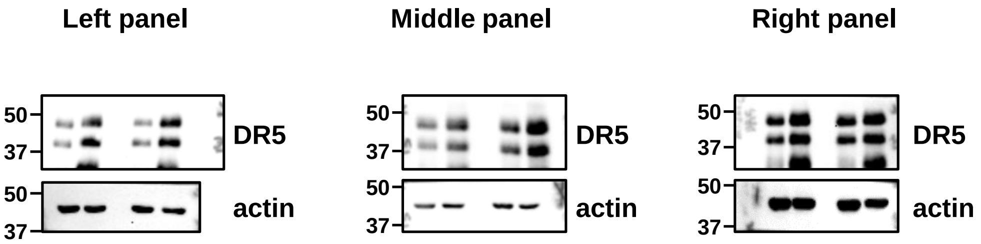
Left panel
Middle panel
Right panel
50
37
DR5
50
37
actin
50
37
DR5
50
37
actin
50
37
DR5
50
37
actin
