# Supplementary material for: Lucanthone, Autophagy Inhibitor, Enhances the Apoptotic Effects of TRAIL through miR-216a-5p-Mediated DR5 Upregulation and DUB3-Mediated Mcl-1 Downregulation
Source: Int J Mol Sci. 2021 Dec 21;23(1):17. doi: 10.3390/ijms23010017 (PMC8744864; doi:10.3390/ijms23010017)
Supplement: Supplementary file 1 [file ijms-23-00017-s001.zip › f-Supplementary figure.pdf]

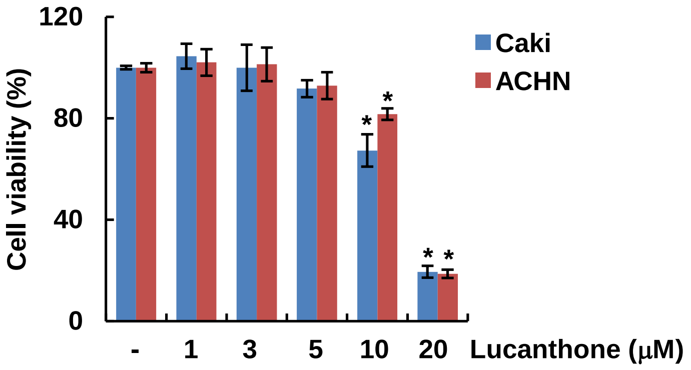

Figure S1. Effect of lucanthone on apoptosis in human renal carcinoma cells. Caki and ACHN cells were treated with 1-20 μM lucanthone, and measured cell viability using XTT assay.

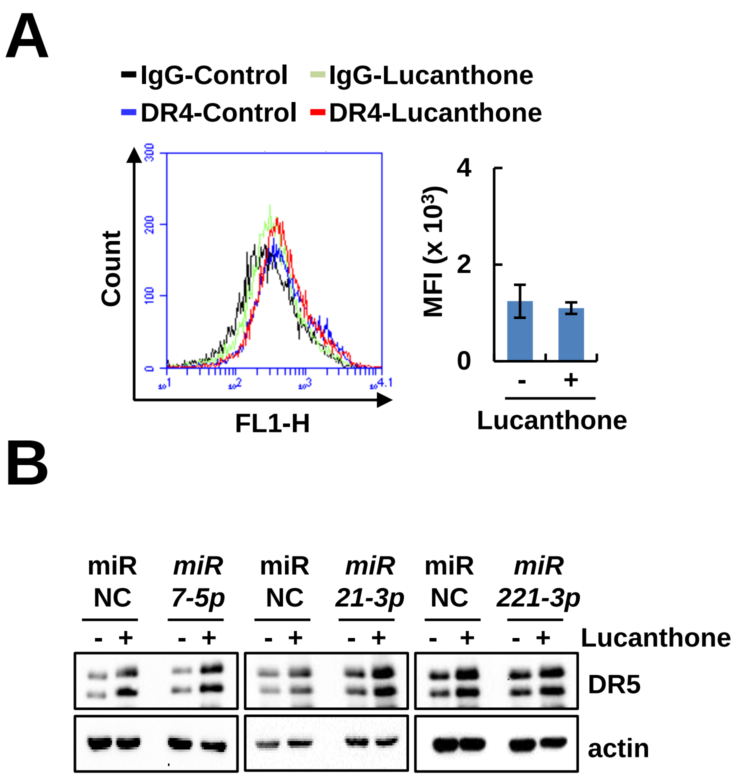

Figure S2. Effect of microRNA mimics on lucanthone-mediated DR5 upregulation. (**A**) Caki cells were treated with 5 μM lucanthone for 24 h, and analyzed DR4 expression on the cell surface using flow cytometry. (**B**) Caki cells were transfected with various microRNA mimics (miR-negative control (NC), *miR-7-5p*, *miR-21-3p*, or *miR-221-3p*) and then treated with 5 μM lucanthone for 24 h. The protein expression was measured by Western blotting. The values in graph (**A**) represent the mean ± SD of three independent samples.
